# Philadelphia County Medical Society

**Published:** 1879-09

**Authors:** 


### PHILADEDPHIA COUNTY MEDICAL SOCIETY.

A conversational meeting was held at the hall of the College of Physicians, Philadelphia, May 14, 1879, Dr. John H. Packard, Vice-President of the Society, occupying the chair.

Dr. Carl Seiler made some remarks on new methods of microscopic investigations. He remarksd that it is of the greatest advantage, in examining tissues with the microscope, to have as large a section as possible, and especially is this the case in pathological histology. He cited a case in which a tumor had been examined by one observer and pronounced to be a fibroma, while another observer had found it to be an epithelioma. This discrepancy was caused by the two observers examining sections made from different parts of the growth, which proved to be an epithelioma when a section of the entire tumor was examined. The Doctor illustrated this part of his remarks by exhibiting an instrument devised by himself, for cutting large and thin sections of tissues, and which was known as Seiler's mechanical microtome. He then went on to explain the methods of hardening tissues preparatory to cutting sections, and gave alcohol the preference over all other hardening agents, if it is used in a week solution at first, and is gradually increased in strength until the tissue is thoroughly hardened.

He next alluded to the different methods of staining the sections so as to differentiate the histological elements, describing the method employed by him for staining with lilac fluid and fixing, subsequently finishing the hardening by strong alcohol. When strong solutions of chromic acid were used, the outer portions of the piece of tissue were quickly hardened, preventing a further penetration of the liquid into the tissue, and in consequence the interior remained soft and in time decayed, making it thus unfit for microscopic examination.

Dr. Charles K. Mills inquired whether the use of alcohol for hardening specimens, and of acids for other purposes mentioned, might not in some cases so change the specimens histologically so as to interfere with their proper examination.

He was of the opinion that large sections are of great advantage, more particularly in studying certain diseases of the spinal cord or brain. In spinal sclerosis we can, by Dr. Seiler's method, readily obtain sections and study the progress and appearance of diseased processes in the different columns of the cord. By this method also large sections can be made of special regions, as the floor of the fourth ventricle, for instance, which is sometimes desirable. Large sections can also be used in determining the physiology and pathology of certain regions of the brain, thus contributing to our knowledge of the localization of functions in the cerebral cortex; for example, the relations to each other of, and the arrangements of elements in, the five or six layers which are known to constitute the gray cortical matter, can be readily investigated. It is well known that large sections of both spinal and cerebral tissue have frequently been made in the old way by skillful operators; but Dr. Seiler here presents us with a time-saving method universally available.

Dr. Nancrede wished to testify to the extreme value of the method which Dr. Seiler has introduced. The case referred to by the lecturer in connection with a section which he exhibited was a most instructive one. A patient had an epithelioma of the lip, which was removed by Dr. Mears. Two years later the patient applied for admission into the Episcopal Hospital, with a tumor under the lower jaw, which the speaker removed, together with part of the bone. The specimen was examined by a committee, who pronounced the tumor a fibroid, and in the specimen they exhibited to the speaker the structure was made up decidedly of fibrous tissue. He came to the conclusion that it was one of the rare cases of fibroid cancer, only two of which are on record, these both having been reported by Paget. In an examination of the tertiary growth, finding evidences of epithelioma, it appeared as if it might be a still rarer instance of fibroid cancer, being interchangeable with epithelioma, and that the terms fibroid cancer and epithelioma are synonymous. If a section such as shown by Dr. Seiler, of the entire growth, had been examined, it would have been all clear, where the epithelioma is very evident at one extremity, but at the other is seen normal fibrous tissue.

Had this subsequeut examination not been made, this case would have been regarded and subsequently quoted as a very unusual and peculiar case, whereas it was only a typical epithelial growth of secondary development. Suspecting that there was some mistake, Dr. Nancrede took the original imbedded portions from which the sections had been removed by the committee, and, leveling the surface of one, to his astonishment found in all his sections true epithelioma, thus showing that the first sections were not through the morbid tissues.

Dr. Seiler, in reply to Dr. Mills, said that alcohol, if used in the manner described, like all other hardening agents, such as chromic acid, picric acid, arsenic acid, etc., acts by dehydrating the tissue and by coagulating the albumen, thus producing no chemical change in that tissue. A shrinking, however, takes place, and especially if the alcohol be used too strong at first; but this is so slight, if the alcohol is gradually increased in strength, that no material difference can be detected in sections made from tissues hardened in this way from those made by the freezing microtome from the fresh tissue. The mineral acid used in removing the excess of color in the stained section, although a rather strong solution, has no other effect upon the tissue itself than to coagulate the albumen still further; and sections examined before the use of the acid, as compared to those after the application of the acid, also show no difference, even under high powers. In regard to brain-tissue, he said that the chromic acid, in the hardening agent known as Muller's fluid, was preferable to alcohol to a certain degree, and that he was in the habit of hardening brain, when large sections were to be made, by immersing the piece of brain in Muller's fluid for a few days, the coloring to be done with dilute hydrochloric acid.

Dr. Seiler next described his new method of double staining. He said that by the carmine only the nuclei of the cells were retained, while all the other histological elements remained colorless; but that these latter could be colored in different tints by a very dilute alcoholic solution of sulph-indigotate of sodium, which had the great advantage of giving a peculiar tint of either green or blue to certain tissues, which was so constant that these tissues could, by their color alone, always be recognized. This double staining, therefore, formed a very important aid in diagnosis, in illustration of which the doctor cited another case of a tumor whose true nature was clearly made out only by the aid of double staining.

During the course of his remarks he passed around, for inspection among the members, very large sections, both single and double-stained, of various tissues, among others a section of the entire adult human larnyx through the vocal cords, measuring one inch by one inch and a half; a longitudinal section of the leg and foot of a five months fœtus, measuring one inch and one-third by three-quarters of an inch, and of extreme thinness and evenness, which were made by his new section-cutter.

THE NASAL DOUCHE.

Dr. Carl Seiler then made some remarks on the use of the nasal douche. He said that many practitioners had given up the use of the douche in the treatment of diseases of the nasal cavity, because they had found inflamations of the Eustachian tube and middle ear, as well as other unpleasant results, to follow the use of this instrument. Dr. Elsberg, of New York, and Dr. J. Solis Cohen, of this city, as well as himself, had but rarely met with a case in which any unpleasant results could be attributed to the use of the nasal douche, although these gentlemen employed this instrument constantly in their practice. He thought that the trouble was to be looked for in a want of attention to certain rules in the use of the nasal douche, which unfortunately, were not generally known or appreciated by the profession.

These rules were: First, that the liquid used should be of the temperature of the body; second, that it should be of the same specfiic gravity as the serum of the blood, to prevent osmosis between it and the blood; a liquid of such density could easily be obtained by dissolving fifty-six grains of common table-salt in a pint of water; and, third, that the bottom of the vessel should not be elevated above the forehead of the patient using it, as otherwise the pressure is too great, and forces the liquid into the frontal sinuses. If used in this way, he felt sure that no evil consequences would be observed following the use of the instrument.

He exhibited several forms of nasal douche, and pointed out their merits and demerits, giving preference to a plain tin cup with a tube attached to the bottom, which is affective, and, at the same time, so cheap as to be within reach of even the poorest classes. A capsule was also exhibited which was made of gelatin, and held the proper quantity of salt to be used, so as to overcome the difficulty and inconvenience to the patient of weighing or measuring the salt every time the douche is to be used.

He, in conclusion, said that he was making experiments with other substances, astringent as well as disinfectant, besides the salt in the nasal douche, but had as yet not come to any definite conclusion in regard to the advisability of using such reagents.

Dr. S. D. Risley said that he had paid no attention to the density of liquids applied to the mucous membranes. It was a new idea to him, and one for which he desired to thank Dr. Seiler. He was disposed to try it in the future, since there was much to be desired in our treatment of mucous surfaces, and this device may prove a valuable addition to our therapeutics. In directing various applications to the mucous membranes, he had done so from other theoretical considerations than osmosis. He had long since given up the nasal douche. believing it did far more harm than good. The osmosis of liquids, as presented by the lecturer, had not occurred to him as a cause for the bad results following its use. Nor had he ceased to use it from any fear that harm would come to the ears from its use. He had never seen any evil resulting to the middle ear from the liquids flowing into the tympanum through the Eustachian tube, and believed that the danger must be very much overstated. The only time he had ever witnessed this accident or knew of its occurrence was while using the posterior nasal syringe in his own hands. The patient was a young man, with enormously hypertrophied tonsils, under treatment for catarrhal deafness. A drop of the solution fell from the syringe into the larynx, causing a sudden and explosive cough. The syringe was emptied at the same instant, a part of its contents passing into the right tympanum, causing great pain and an extensive rupture of the membrane of the tympanum, which, however, healed rapidly and did no permanent harm.

While the abnormal situation of the orifice of the Eustachian tube might facilitate the entrance of fluids, as pointed out by Dr. Cohen, he thought the real necessity for the precaution not to swallow while using the nasal douche had not been mentioned. In the act of swallowing, the orifices of the tubes are opened, and thus the entrance of air or liquid is facilitated. The otologist is constantly taking advantage of this fact in treating disease of the middle ear. The nasal douche did harm very frequently, because in a very large number of cases relatively of nasopharyngeal catarrh the disease was not general until made so by the application of medicated liquids to healthy mucous membrane. He was convinced that in a very large number of cases the disease was a local one, originally set up, it might be, by a foreign body lodged in the nasal passages, or by some anatomical peculiarity bringing parts into contact which are normally separated, producing localized ulceration in the mucous membrane. In such cases the douche could be of no service further than to cleanse the nose and pharynx occasionally, the ulcer meantime being treated by topical applications. Medicated solutions applied by means of the douche must necessarily affect the healthy mucous membraue, sooner or later bringing on the chronic granular inflamation and profuse muco-purulent discharge met with in these cases of naso-pharyngeal disease. It remains to be seen whether the expedient suggested by Dr. Seiler will relieve the douche of the distrust which many feel regarding its value as a therapeutic measure.

Dr. S. Solis Cohen said that in his experience difficulty following the use of the nasal douche is generally due to the fact that the water has been used too cold. There is one point in this connection that is not well understood. The locality of the opening of the Eustachian tube varies in different persons, and the opening is of different shapes, being sometimes narrow and sometimes trumpet-shaped. In most of the cases where there is trouble produced by the use of the douche, this opening is very patent and is situated unusually low down.

Patients should be instructed not to swallow while using the douche, as the liquid may be forced into the open Eustachian tube and into tne middle ear.

The speaker does not use the douche to carry medicaments into the nasal passages and pharynx, but simply for the purposes of cleansing. The ordinary ball-aud-tube syringe makes a good douche by starting the fluid with the bulb and elevating the basin above the head, when the water will continue to flow. It is a very good precaution, also, to observe, to have the water boiled that is to be used in the douche or as a gargle, which will precipitate and remove many of the impurities of the ordinary river-water.

Dr. C. Seiler agreed with Dr. C. Cohen, that the douche should, as a rule, be used only for cleansing. He had been experimenting, however, with some cases, and was not prepared to assert that there might not be some exceptions. Some cases have done very well under astringent injections.

In regard to Dr. Risley's remarks, he would say that he was aware that an ulcerated condition of the mucous membrane often exists requiring examination and topical applications. The douche is not curative in itself, but must be accompanied by local measures. In some cases, where there has been dryness of the mucous membrane without any special lesion, injections of the density and temperature of the blood, containing gr. x nitrate of silver solution, had been of great service. He did not believe that a medicated solution of this character could be injurous to healthy mucous membrane, although it would stimulate the diseased surfaces.—*Phila. Med. Times.*

